# Laparoscopic transmesenteric pyeloplasty and isthmusectomy for adult horseshoe kidney with recurrent symptomatic hydronephrosis

**DOI:** 10.1002/iju5.12192

**Published:** 2020-07-21

**Authors:** Li Lu, Bo Ma, Wenwen Zhong, Bin Yao, Dejuan Wang, Jianguang Qiu

**Affiliations:** ^1^ Department of Urology the Sixth Affiliated Hospital of Sun Yat‐sen University Guangzhou Guangdong China

**Keywords:** horseshoe kidney, isthmusectomy, laparoscopic, pyeloplasty, transmesenteric

## Abstract

**Introduction:**

There is no consensus for the horseshoe kidneys with recurrent symptomatic hydronephrosis, so we presented our initial experience with a laparoscopic transmesenteric approach.

**Case presentation:**

Five patients with symptomatic ureteropelvic junction obstruction were identified by computed tomography urography. The laparoscopic transmesenteric approach was performed in such a way that the mesentery of the small intestine was incised along with the blood vessels longitudinally, and the isthmus was isolated to avoid injury to the mesenteric blood supply; then we cut the isthmus using an endostapler. The ureteropelvic junction obstruction was removed via the Anderson‐Hynes technique. The operation time was 135–204 min, and the estimated blood loss was 50–120 mL. Patients had a 5.7‐day stay postoperatively, there were no other injuries or complications, and ultrasound scans or computed tomography urography showed good postoperative effects.

**Conclusion:**

Laparoscopic transmesenteric surgery is a feasible and safe procedure for selected cases with horseshoe kidney with recurrent symptomatic hydronephrosis.

**Abbreviations & Acronyms**
CTU: computed tomography urography
UPJO: ureteropelvic junction obstruction

**Keynote message**

Horseshoe kidneys are associated with UPJO, and there is a lack of consensus on the treatment for adult patients. Laparoscopic and robotic surgeries have been successful for the treatment of horseshoe kidneys with UPJO via a retroperitoneal approach, although the number of patients studied is limited. Here, we report our initial effective use of laparoscopic transmesenteric surgery to treat horseshoe kidneys with recurrent symptomatic hydronephrosis.

## Introduction

The horseshoe kidney is a congenital urological anomaly, estimated to occur in 1/400 of the population. UPJO is the most frequent complication in approximately one‐third of patients.^1^ The basic treatment for UPJO is the resection of the strictured segment and inserting the ureter into the dependent part of the pelvis.^2^ Treatment strategies include palliative interventions such as percutaneous nephrostomy or antegrade ureteric stenting, but surgery is more definitive, including open urethroplasty or laparoscopic pyeloplasty.^3^ Isthmusectomy is also an effective method that results in as close to physiological kidney drainage as possible,^4^ but it is difficult to perform due to the increased risk of complications, including bleeding and collateral kidney infarction, especially in patients with a previous surgical history or recurrent symptomatic hydronephrosis.^5^

Previous reports on laparoscopic transmesenteric pyeloplasty in children with horseshoe kidneys have shown acceptable, favorable results, although the number of cases is limited.^6^ However, there have been no consensus on the presence of a horseshoe kidney with recurrent symptomatic hydronephrosis. In this study, we present our experience with an operative technique and primary outcomes.

## Case presentation

**Fig. 1:**
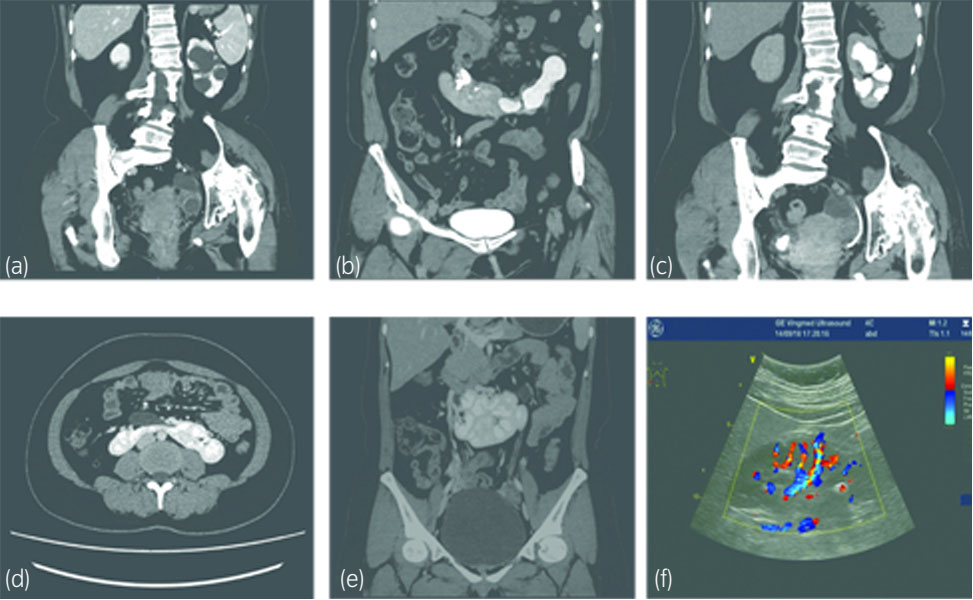
Preoperative CT scan of the horseshoe kidney and postoperative follow‐up image (Case no. 3, a, b: preoperative CTU, c: postoperative CTU at 8 weeks; Case no. 5, d, e: preoperative CTU, f: ultrasound image at 8 weeks).

All five patients, including four males and one female ranging in age from 21 to 43 years (mean 32.8), were admitted to our center for complaining of flank pain (*n* = 3) or urinary infection with microhematuria (*n* = 2); the mean age was 32.8 years (range 21–43). Three patients were diagnosed with hydronephrosis UPJO: one female patient had left severe hydronephrosis with coexisting kidney stones, and other two patients had recurrent urinary infections for several years. The thickness of the isthmus was analyzed using CTU (Fig. 1a,b,d,e), and renal function testing was carried out with 99mTc‐DTPA.

All patients were eligible for laparoscopic surgery. Before the surgical procedure, cases with urinary infections undergone the urine culture and treated with antibiotic drug. Patients were placed in the supine position with a 10‐cm pad on the flank, and all procedures were completely successful with the use of a transmesenteric approach and four trocars (2 × 5 mm and 2 × 10 mm).

**Fig. 2:**
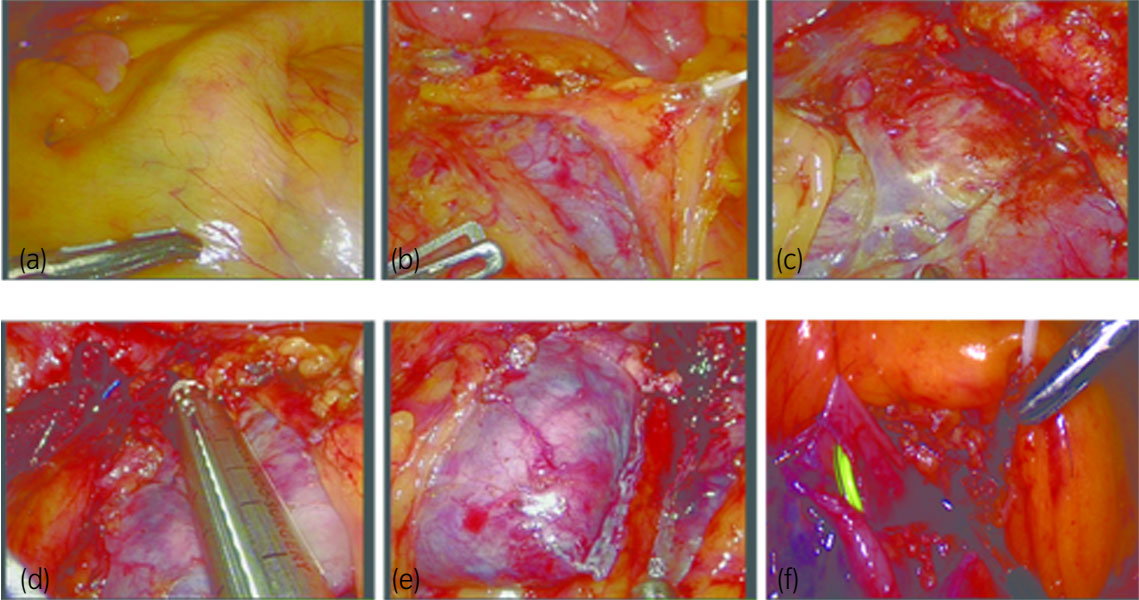
Pictures of the surgical procedure (a: exposure of the mesentery; b: thorough incision of the mesentery; c: blood supply of the isthmus; d, e: isthmusectomy using an endostapler; f: pyeloplasty).

1. First, the intestine was rolled and suspended with a hanging and fastening method,^7^ and thorough dissection of the mesenteric fascia was performed to avoid injuring the blood vessels and intestine (Fig. 2a,b). The ascending portion of the duodenum was visualized moving along the ascending colon.
2. The isthmus was isolated and then separated from the fascia of the infrarenal abdominal aorta, and the prepared blood vessels were separated from the surrounding tissues. After this, the vessels that reached the lower pole and isthmus were identified. The large blood vessels that supply the lower pole of the isthmus were pulled away (Fig. 2c); then the tiny blood vessels leading to the isthmus were clipped or coagulated if there was not a high risk of kidney ischemia.
3. Finally, the isthmus was raised upward, and the laparoscopic endostapler was placed under the isthmus. After separating the isthmus, if necessary, the edges of the isthmus on both the upper and lower sides were sutured with Vicryl 2‐0 (Fig. 2d,e). During this step, we considered that bipolar coagulation was not suitable for hemostasis.
4. Pyeloplasty was initiated in two patients according to Anderson‐Haynes. We achieved exposure of the UPJO using the 4‐point suspension fixation technique, and the pelvis was anastomosed with the ureter according to the Anderson‐Hynes dismembered pyeloplasty procedure (Fig. 2f).

All patients were discharged within 5.7 days (range 4–8 days), and no bowel disorders were found. All double‐J stents were removed 4 weeks postoperatively, and the patients were evaluated via ultrasonography 1 month later; all patients were treated successfully within the follow‐up period (11–25 months).

## Discussion

Among patients with horseshoe kidney, the occurrence rate of hydronephrosis was 14% to 35% approximately; this presentation differs from the clinical presentation in children. Calculi, nephrocarcinoma, and polycystic disease followed by UPJO are the major pathological entities of the disease, so most patients require surgery.^8^ There remains a debate on the choice of surgical procedure used for UPJO in cases of horseshoe kidney. As the ureters are located anterior to the isthmus, which often results in hydronephrosis, dismembering pyeloplasty should be the procedure of choice.^9^ Isthmusectomy allows separation of the horseshoe kidneys with a more dependent position, hence providing the benefit of urine drainage and decreasing the risk of obstruction due to the maintenance of the newly reconstructed ureteropelvic site.^10^

The retroperitoneal laparoscopic approach has been the primary choice in most institutions; it has the theoretical advantage of preventing intra‐abdominal adhesions, but CO_2_ is difficult to confine to the retroperitoneal space while maintaining an adequate space for this procedure.^11^ Our initial experience showed that the transperitoneal and transmesenteric approaches combined with the hanging and fastening method could provide adequate space and a clear visual field during surgery; all patients demonstrated increases in or maintenance of differential renal function postoperatively. In conclusion, we believe that our modification could be a technique of choice for the experienced surgeon with the UPJO in the adult horseshoe kidney.

## Acknowledgment

This study was funded by the Natural Science Foundation of Guangdong Province (Dr. Li Lu, 2017A030310208).

## Conflict of interest

None declared.
